# Risk Factors Associated with Uterine Rupture and Dehiscence: A Cross-Sectional Canadian Study

**DOI:** 10.1055/s-0041-1739461

**Published:** 2021-12-06

**Authors:** Ernesto Antonio Figueiró-Filho, Javier Mejia Gomez, Dan Farine

**Affiliations:** 1 Department of Obstetrics and Gynecology, Division of Maternal-Fetal Medicine, Mount Sinai Hospital, University of Toronto, Toronto, ON, Canada; 2 Department of Obstetrics and Gynecology, Mount Sinai Hospital, University of Toronto, Toronto, ON, Canada

**Keywords:** urupture, dehiscence, relative risk, cross-sectional, perinatal outcomes, ruptura uterina, deiscência, risco relativo, transversal, resultados perinatais

## Abstract

**Objective** To compare maternal and perinatal risk factors associated with complete uterine rupture and uterine dehiscence.

**Methods** Cross-sectional study of patients with uterine rupture/dehiscence from January 1998 to December 2017 (30 years) admitted at the Labor and Delivery Unit of a tertiary teaching hospital in Canada.

**Results** There were 174 (0.1%) cases of uterine disruption (29 ruptures and 145 cases of dehiscence) out of 169,356 deliveries. There were associations between dehiscence and multiparity (odds ratio [OR]: 3.2; *p* = 0.02), elevated maternal body mass index (BMI; OR: 3.4; *p* = 0.02), attempt of vaginal birth after a cesarian section (OR: 2.9; *p* = 0.05) and 5-minute low Apgar score (OR: 5.9; *p* < 0.001). Uterine rupture was associated with preterm deliveries (36.5 ± 4.9 versus 38.2 ± 2.9; *p* = 0.006), postpartum hemorrhage (OR: 13.9; *p* < 0.001), hysterectomy (OR: 23.0; *p* = 0.002), and stillbirth (OR: 8.2; *p* < 0.001). There were no associations between uterine rupture and maternal age, gestational age, onset of labor, spontaneous or artificial rupture of membranes, use of oxytocin, type of uterine incision, and birthweight.

**Conclusion** This large cohort demonstrated that there are different risk factors associated with either uterine rupture or dehiscence. Uterine rupture still represents a great threat to fetal-maternal health and, differently from the common belief, uterine dehiscence can also compromise perinatal outcomes.

## Resumo

**Objetivo** Comparar os fatores de risco maternos e perinatais associados à ruptura uterina completa e deiscência uterina.

**Métodos** Estudo transversal de pacientes com ruptura/deiscência uterina no período de janeiro de 1998 a dezembro de 2017 (30 anos) internadas na Unidade de Parto de um hospital universitário terciário no Canadá.

**Resultados** Ocorreram 174 (0,1%) casos de transtorno uterino (29 rupturas e 145 deiscências) em 169.356 partos. Houve associações entre deiscência e multiparidade (razão de chances [RC]: 3,2; *p* = 0,02), índice de massa corporal (IMC) materno elevado (RC: 3,4; *p* = 0,02), tentativa de parto vaginal após cesariana (RC: 2,9; *p* = 0,05) e baixa pontuação Apgar em 5 minutos (RC: 5,9; *p* < 0,001). A ruptura uterina foi associada a partos prematuros (36,5 ± 4,9 *versus* 38,2 ± 2,9; *p* = 0,006), hemorragia pós-parto (RC: 13,9; *p* < 0,001), histerectomia (RC: 23,0; *p* = 0,002) e natimorto (RC: 8,2; *p* < 0,001). Não houve associação entre ruptura uterina e idade materna, idade gestacional, início do trabalho de parto, ruptura espontânea ou artificial de membranas, uso de ocitocina, tipo de incisão uterina e peso ao nascer.

**Conclusão** Esta grande coorte demonstrou que existem diferentes fatores de risco associados à ruptura ou à deiscência uterina. A ruptura uterina ainda representa uma grande ameaça à saúde materno-fetal e, diferentemente da crença comum, a deiscência uterina também pode comprometer os desfechos perinatais.

## Introduction

Uterine rupture is defined as complete disruption of all uterine layers during pregnancy, delivery, or immediately after delivery. It is a catastrophic situation in obstetrics, and, although rare, often results in both maternal and fetal adverse consequences. 1 2 3 Uterine rupture can be complete or partial (dehiscence). Complete rupture usually involves direct communication between the uterine cavity and the peritoneum, and is associated with high rates of perinatal mortality and morbidity. 1 2 3 Dehiscence presents when the myometrium is covered by the visceral peritoneum, often an incidental finding in caesarean deliveries, and usually described without any major medical complications. 4 5

The incidence of uterine rupture ranges between 0.5 and 5.3 per 10 thousand deliveries, 6 7 and mostly happens during trial of labor after a cesarian section (TOLAC). 7 8 Uterine rupture is also described in women without a previous cesarian section, during spontaneous onset of labor. 9 10 11 In the Netherlands, this incidence is of around 0.007%. 10 Lower incidences were also reported in the United States (1/16,849) 12 and in the United Kingdom (0.2/1,000). 11

The prevalence of uterine rupture tends to be lower in developed countries. 7 The risk factors include prior uterine scar 13 and the use of uterotonics. 14 Other risk factors associated with uterine rupture include inappropriate induction/augmentation of labor, obstructed labor, previous uterine trauma, grand multiparity, abnormal placentation, fetal anomalies, advanced maternal age, high body mass index (BMI), and lack of antenatal care. 2 14 15 16

To provide a better insight into the safety and adequacy of the current obstetrical practice, the identification of certain risk factors becomes crucial for the improvement of healthcare. Thus, the objective of the present study was to identify the perinatal factors associated with uterine rupture or dehiscence in a tertiary high-risk obstetrical care centre in Canada. Another objective was to compare these identified perinatal factors among cases of complete uterine rupture and cases of dehiscence.

## Methods

Cross-sectional study of patients with uterine rupture from January 1998 to December 2017 who were admitted at the Labor and Delivery Unit of a tertiary teaching hospital in Canada. The code of the International Statistical Classification of Diseases, Ninth and Tenth Revisions (ICD-9/ICD-10) was used to identify eligible patients to perform chart reviews. All cases identified by ICD-9/ICD-10 within the 30-year period were included. The Institutional Ethical Review Board approved the study protocol (protocol #18–0099-C).

The clinical information obtained from the chart review included maternal age, parity, BMI, obstetric history (including gestational age at delivery, type of previous cesarean section incision, onset and manifestation at rupture, delivery method, maternal complications), and neonatal outcomes (birthweight, Apgar score at 1 and 5 minutes, admission to the Neonatal Intensive Care Unit [NICU], and stillbirth). Known risk factors for uterine rupture, including advanced maternal age, multiparity, and TOLAC were also collected during the chart review. The lower-segment uterine scar was defined as previous low transverse cesarean section, while the non-lower-segment scars were those with classical cesarean section and other uterine surgeries, either through laparoscopy or laparotomy.

Uterine rupture was defined as complete disruption of all layers of the uterus - the endometrium, myometrium and perimetrium. 17 Uterine dehiscence was defined by incomplete division of the uterine wall that does not encompass all uterine layers. 18 Uterine dehiscence can also cause the thinning of the uterus, often allowing the fetus to be seen through the myometrium. 18

The data was tabulated in Microsoft Excel 2007 (Microsoft Corp., Redmond, WA, United States worksheets connected to the R (R Foundation for Statistical Computing, Vienna, Austria) software, and compiled into double-entry contingency tables. The statistical analysis was performed using Fisher Exact Test or the Chi-squared Test with Yates correction. Associations were considered statistically significant when *p* ≤ 0.05. Odds ratios (ORs) were calculated between associations with Confidence Interval of 95% (95%CI). The Student *t* -test was used to compare the means of parametric variables between the groups, with results expressed as means and standard deviations. For the other variables, the simple proportion test was used, with values expressed as a percentage.

## Results

During the studied period (1988–2017), we found 174 cases of uterine disruption (0.1%; 29 complete uterine ruptures and 145 cases of dehiscence) out of 169,356 deliveries. Cesarean section was performed for most cases of uterine dehiscence (121/145; 83%) and uterine rupture (26/29; 90%). Among all the patients who presented with uterine disruption ( *n* = 174), the mean maternal age was 34 ± 4.5years, the average gestational age was 38 ± 3.3weeks, with a maternal BMI of 26.1 ± 6.7kg/m ^2^ . The mean neonatal birthweight was 3291 ± 771 g. There were no statistically significant differences between the groups, except that pregnant women who presented with complete uterine rupture delivered more preterm neonates compared with those who had dehiscence (36.5 ± 4.9 vs 38.2 ± 2.9; *p* = 0.006).

Table 1 outlines demographics, management and outcomes associated with uterine rupture and dehiscence. In the demographics, there were some variables that were statistically different between the two groups, such as multiparity (OR: 3.2; *p* = 0.02) and elevated maternal BMI (OR: 3.4; *p* = 0.02). Neither uterine rupture nor dehiscence would be predictive for diagnosing. No associations were identified regarding complete uterine rupture or dehiscence and maternal age, gestational age at delivery, and onset of labor.

**Table 1:** Associations between uterine rupture, dehiscence, and maternal and perinatal risk factors identified during the study period

Variables	Uterine disruption			Odds ratio (95% confidence interval)	*p* -value
	Dehiscence n = 145	Rupture n = 29	Total n = 174		
*Maternal age (years)*					
> 35	76 (44%)	18 (10%)	94 (54%)	1.5	0.34
< 35	69 (40%)	11 (6%)	80 (46%)	(0.7–3.4)	
*Parity*					
> 2	18 (10%)	9 (5%)	27 (15%)	3.2	0.02**
< 2	127 (73%)	20 (12%)	147(85%)	(3.2–1.3)	
*Maternal body mass index*					
> 30	12 (7%)	7 (4%)	19 (11%)	3.4	0.02**
< 30	133 (76%)	22 (13%)	155 (89%)	(1.2–9.5)	
*Gestational age at delivery (weeks)*					
< 37	17 (10%)	7 (4%)	24 (14%)	2.4	0.13
> 37	128 (73%)	22 (13%)	150 (86%)	(0.9–6.4)	
*Onset of labor*					
Spontaneous	36 (21%)	11 (6%)	47 (27%)	1.9	0.14
Induction	109 (63%)	18 (10%)	127 (73%)	(0.8–4.3)	
*Use of oxytocin*					
Yes	28 (16%)	10 (6%)	38 (22%)	2.2 (0.9–5.2)	0.07
No	117 (67%)	19 (11%)	136 (78%)		
*Trial of labor after cesarian section (TOLAC)*					
Yes	12 (7%)	6 (3%)	18 (10%)	2.9	0.05**
No	133 (77%)	23 (13%)	156 (90%)	(1.0–8.5)	
*Spontaneous rupture of membranes > 6h*					
Yes	45 (26%)	10 (6%)	55 (32%)	1.2	0.71
No	100 (57%)	19 (11%)	119 (68%)	(0.5–2.7)	
*Artificial rupture of membranes*					
Yes	77 (44%)	12 (7%)	89 (51%)	0.6	0.24
No	68 (39%)	17 (10%)	85 (49%)	(0.3–1.4)	
*Type of incision*					
Low transverse	89 (51%)	16 (9%)	105 (60%)	0.8	0.53
Classic/Other	56 (32%)	13 (8%)	69 (40%)	(0.3–1.7)	
*Postpartum hemorrhage*					
Yes	7 (4%)	12 (7%)	19 (11%)	13.9	< 0.001**
No	138 (79%)	17 (10%)	155 (89%)	(4.82–40.1)	
*Hysterectomy*					
Yes	1 (1%)	4 (2%)	5 (3%)	23.0	0.002**
No	144 (83%)	25 (14%)	169 (97%)	(2.5–214.7)	
*Neonate birthweight*					
> 4,000 g	22 (13%)	5 (3%)	27 (16%)	1.2	0.99
< 4,000 g	123 (70%)	24 (14%)	147 (84%)	(0.4–3.4)	
Apgar score (at 5 minutes)					
< 4	25 (14%)	16 (9%)	41 (23%)	5.9	< 0.001*
> 5	120 (70%)	13 (7%)	133 (77%)	(2.5–13.8)	
*Stillbirth*					
Yes	0 (0%)	9 (5%)	9 (5%)	8.2	< 0.001**
No	145 (83%)	20 (12%)	165 (95%)	(5.5–12.4)	

Notes: Percentages are expressed in relation to the total number of cases ( *n* = 174); *Pearson Chi-Square Test; **Fisher Exact Test.

Management of labor did not differ statistically in the use of oxytocin, artificial rupture of membranes, and type of uterine incision, whereas there was a significant association between dehiscence and TOLAC (OR: 2.9; *p* = 0.05). The outcomes of uterine rupture were much worse than those of dehiscence, with postpartum hemorrhage (OR: 5.8; *p* < 0.001), hysterectomy (OR: 5.4; *p* = 0.002), and stillbirth (OR 8.3; *p* < 0.001). Interestingly, low 5-minute Apgar scores were more frequently associated with dehiscence (OR: 5.9; *p* < 0.001).

## Discussion

The present retrospective 30-year cohort described the incidence of uterine rupture in one of the busiest hospitals in Canada. Among the charts analyzed, 83% (145/174) of the cases were of dehiscence, and 17% (29/174) were of complete uterine rupture with either maternal or fetal adverse outcomes associated. There were significantly more maternal hysterectomies (5%), PPH (11%), lower neonatal 5-minute Apgar score (23%), and stillbirth (5%) related with complete uterine ruptures.

Maternal hemorrhage, blood transfusion, and hysterectomy are the major maternal risks associated to uterine rupture. 1 14 19 Maternal hemorrhage rates associated to uterine rupture range from 1.2% to 13.8%. 5 20 21 In our series, we identified 19/174 (11%) cases of postpartum hemorrhage (PPH), mostly (12/174; 7%) from major ruptures with elevated OR (13.9; 95%CI: 4.8–40.1; *p* < 0.001). Hysterectomy was associated to uterine rupture in 5/174 (3%) cases, which resulted in an OR of 23.0 (95%CI: 2.5–214.7; *p* = 0.002). Our findings were not different from those of Barger et al. (2011), 22 who evaluated severe outcomes associated with uterine rupture, including PPH and hysterectomy in 14% of studied mothers.

We also identified an association between multiparity (> 2) (27/174 [15%]; OR: 3.2; 95%CI: 3.2–1.3; *p* = 0.02) and high BMI (> 30kg/m ^2^ ) (19/174 [11%]; OR: 3.4; 95%CI: 1.2–9.5; *p* = 0.02) with uterine rupture in our series. Similar results were described by Al-Zirqi et al. (2016), 14 who identified a 2.4-fold increase in the odds of uterine rupture in multipara (> 3). Another study 23 described that TOLACs in obesity pregnancies (BMI > 30Kg/m ^2^ ) at term increase the risk of maternal (blood transfusion, uterine rupture, admission to the Intensive Care Unit [ICU]) and neonatal complications (low 5-minute Apgar score, NICU admission, neonatal death). Our study did not find associations between uterine rupture and maternal age, gestational age at delivery, and membranes status, as opposed to other studies found in the literature. 1 24

Among the fetal/neonatal complications associated with uterine rupture, our 30-year case review demonstrated elevated relative risk (RR) of stillbirth (9/174; 5%) (OR: 8.2; 95%CI 5.5–12.4; *p* < 0.001). The risk of perinatal death associated with uterine rupture was of 8.7% in a population-based cohort in the Netherlands. 10 Another similar study 25 demonstrated a rate of 0.4 perinatal deaths per 1,000 associated with uterine ruptures during a 20-year period. Several factors are associated to neonatal injury in the context of uterine rupture, and it depends on the severity of the rupture, placenta site, preexisting fetal comorbidities, and the degree of umbilical cord compression. 19 In our experience, all the stillbirths were associated with complete rupture, although dehiscence was mostly associated with low Apgar scores (25/174; 14%). In a case-control study 26 in Finland, the authors analyzed 197 cases of obstetric near-miss complications, and, similarly to our findings, identified 8 (4%, 8/197) cases of stillbirth, all of them consequences of uterine rupture, though the type (complete or dehiscence) was not described.

It is unclear how accurate our statistics of stillbirth as a consequence of uterine rupture are. The present study raised this question as we could distinguish between a case of dehiscence and one of complete uterine rupture in our series. Regarding the number of stillbirths out of the total cases of uterine disruption (without separation between dehiscence and complete rupture), we found a rate of 5% of stillbirths (9/174), similar to that of the Finnish study (4%, 8/197). Conversely, if we look at the true stillbirth rate of uterine ruptures, it becomes 31% (9/29). A worse perinatal outcome has been described by Berhe et al. (2015) 27 in Ethiopia (44 stillbirths/47 major ruptures; 95%). Berhe et al.'s 27 results reflected a selection of only major uterine ruptures (thus eliminating dehiscences), and, possibly, the fact that the study was performed in a developing country. Among the 9 cases of stillbirth in our series, 7 were due to TOLAC failure, and 2 were due to spontaneous onset of labor with no previous cesarean sections.

A recent study 28 also from Ethiopia reinforced the importance of identifying the risk factors for uterine rupture in specific contexts. The authors compared 135 women with uterine rupture and 270 controls of women without uterine rupture. The risks associated with uterine rupture were poor antenatal care (only one prenatal visit), obstructed labor, and macrosomia. The maternal mortality rate was 9.6%, with 75% of stillbirths. Hysterectomy was performed in 55.6% of mothers, and PPH was demonstrated in 57.8% of the cases.

Although women with previous lower-segment cesarean section who undergo induction of labor are more likely to have uterine rupture than those with previous vaginal deliveries, 29 this association was not found in the present study. We could not find an association between uterine rupture and previous uterine scar or induction/augmentation of labor. In contrast, a recent study in Denmark 9 identified the association between uterine rupture and augmentation of labor in multiparous women. Other studies have also shown that double-layer closure of the uterus in previous cesarean sections, compared with single-layer closure, is associated with a thicker third-trimester lower uterine segment, 30 suggesting that the inclusion of the measurement of the thickness of the lower uterine segment in the decision of the route of delivery 31 can reduce the rates of uterine rupture. We were not able to extract data on lower segment thickness in this series.

We found an increased risk of uterine rupture among the patients who underwent TOLAC (18/174, 10%), with an OR of 2.9 (95%CI: 1.0–8.5; *p* = 0.05), which corroborates findings already described. 29 32 33 A meta-analysis 34 evaluating women with previous cesarian sections (CSs) and the risk of uterine rupture found that vaginal births after cesarian section (VBACs) after 2 previous cesareans were at significantly higher risk of rupture than those with 1 previous CS (1.59% versus 0.72%), with an overall success rate for vaginal delivery of 71.1%.

A great asset of the present study was the fact that, with our data, we could distinguish between complete uterine rupture and dehiscence among the patients included. Our findings contributed to reinforce the importance of the risk factors observed, while counseling patients in obstetrical practice. Some weaknesses were associated to the cross-sectional/observational aspect of the study. It was not possible to access the same variables among the patients who did not present ruptures and dehiscence. Thus, the comparisons were made only between patients who presented complete uterine rupture and those who had dehiscence. We were not able to compare patients with complete uterine rupture and dehiscence with those with intact uterine walls. The convenience sample used was based on the diagnostic coding of the conditions (rupture/dehiscence), therefore only allowing full access to the identified clinical charts. It is also important to mention that bias could not be minimized in the present study, as data were not stratified and multivariate analysis was not applied, due to the convenience aspect of the sample, associated with the low prevalence of uterine rupture.

## Conclusion

In summary, our 30-year analysis of uterine rupture in a Canadian population demonstrated that there are different risk factors associated with complete uterine rupture or dehiscence. We found that multiparity, high maternal BMI, TOLAC, low 5-minute Apgar scores were more associated with dehiscence, whereas preterm deliveries, postpartum hemorrhage, hysterectomy and stillbirth were risk factors more associated with complete uterine ruptures. Even though the information we present corroborates findings already described in literature, this is the first time that the risks are separated and compared between complete uterine rupture and dehiscence. Our study confirms that uterine rupture still represents a great threat to maternal-fetal health and introduces the idea that dehiscence can also challenge maternal and perinatal outcomes.
